# Schwannoma originating in the soft palate: A case report

**DOI:** 10.1016/j.ijscr.2021.106108

**Published:** 2021-06-17

**Authors:** Alhanouf A. Alhedaithy, Anwaar M. Alsayed, Khalid A. Al-Sindi, Waleed M. Janahi

**Affiliations:** a King Fahad Medical Military Complex, Dhahran, Saudi Arabia; b King Hamad University Hospital, Al Muharraq, Bahrain

**Keywords:** Schwannoma, Schwann cell, Soft palate, Immunohistochemical staining, Case report

## Abstract

**Introduction and importance:**

Schwannomas are relatively uncommon, benign, slow-growing neoplasms, which are derived from schwann cells that can arise from any cranial, peripheral, or autonomic nerves. The involvement of the palate is a rare presentation and hardly reported in the literature.

**Case presentation:**

Here, we report the case of a 39-year-old woman with a history of a foreign body sensation in the throat and difficult swallowing.

**Clinical findings and investigations:**

Oral examination showed a smooth, non-tender, right-sided, soft palate mass. Computed tomography (CT) scan revealed a well-defined, non-enhancing, homogenous pedunculated soft tissue mass arising from the posterior edge of the right side of the soft palate.

**Interventions and outcome:**

The mass was excised completely under local anesthesia in the clinical setting using a CO_2_ surgical laser. The mass was sent for histologic analysis, which confirmed the diagnosis of a benign schwannoma.

**Conclusion:**

Eventually, upon follow-up at six months post excision, no evidence of recurrence was detected.

## Highlights

• A 39-year-old woman presented with a two-month history of right sided soft palate mass causing difficult swallowing.
• The mass was completely excised in a clinical setting under local anesthesia using a CO2 surgical laser.
• Histopathologic analysis confirmed the diagnosis of a benign soft palate Schwannoma.
• After six months post excision, no evidence of recurrence was detected.

## 1 Introduction

This work has been reported in line with the SCARE criteria [1].

Schwannomas are slow-growing, benign, soft tissue neoplasms of the neuroectodermal origin. They are derived from schwann cells, which are the sheath cells that cover nerve fibers [2,3,4]. Approximately 25%–45% are seen in the head and neck region including the oral cavity, orbit and salivary glands; however, schwannomas in the soft palate are considered rare with only 1% of schwannomas found within the oral cavity [5,6]. Almost 90% of schwannomas are sporadic in nature while 3% are associated with neurofibromatosis type 2, 2% with schwannomatosis and 5% with meningiomatosis with or without neurofibromatosis type 2. Pain and neurological symptoms are uncommon complaints unless the tumor is large [7]. Schwannomas waxes and wanes in size, which may be related to the amount of cystic degeneration it contains. Essential features of a schwannoma include a biphasic tumor with highly ordered cellular component (Antoni A) that palisades (Verocay bodies) plus myxoid hypocellular component (Antoni B). Additionally, a strong immunoreactivity for S100 protein (A marker for peripheral nerve sheath tumors). Proper investigations, including those that employ histopathologic techniques are essential to confirm the diagnosis of a shwannoma. Surgical excision is the treatment of choice for management with rare recurrence.

In this case report, we describe a 39-year-old female patient with a history of a foreign body sensation in the throat and difficult swallowing. She was found to have schwannoma of the soft palate.

## 2 Presentation of case

A 39-year-old female presented to the outpatient clinic at King Hamad University Hospital in Al Muharraq, Bahrain, complaining of discomfort during swallowing associated with sensation of foreign body in the throat. She first noticed the mass two months prior to presentation. At that time, the mass was painless but gradually increasing in size. She was otherwise healthy and reported no history of smoking or alcohol consumption. No genetic or syndromic abnormalities were reported.

**Fig. 1:**
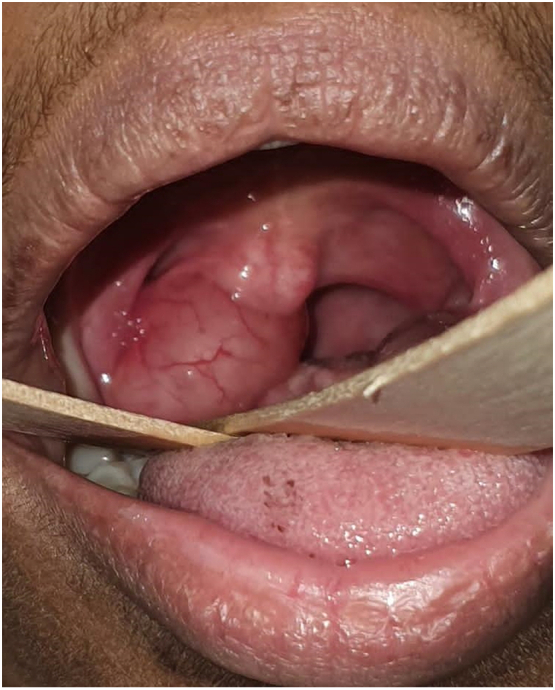
Painless solitary cystic soft tissue lesion on the right side of the soft palate.

**Fig. 2:**
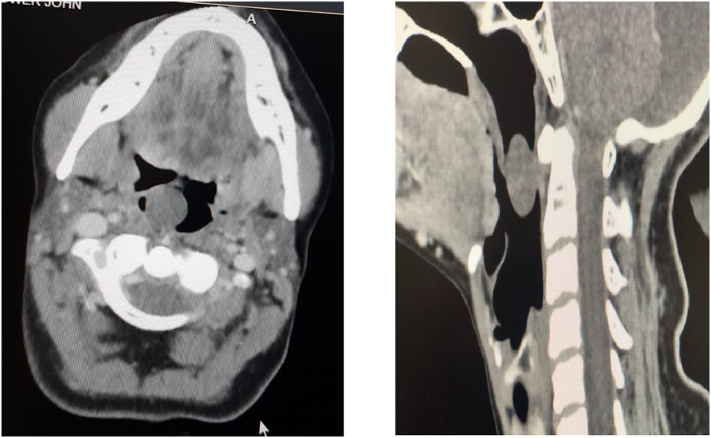
Axial and sagittal CT scans showing a soft tissue lesion arising from the right side of the soft palate.

Clinical examination of the intraoral cavity revealed a solitary, cystic, non-tender soft tissue mass that protruded over the right side of the soft palate (Fig. 1). No regional lymph nodes were palpable. Laboratory reports were unremarkable. Computed tomography (CT) scan was scheduled one week later which revealed a well-defined, non-enhancing, homogenous pedunculated soft tissue mass measuring 2.1 × 1.6 × 1.5 cm arising from the posterior edge of the right side of the soft palate. The mass was protruding into the oral cavity, and the peduncle of the mass had smooth margins (Fig. 2). A treatment option of undergoing complete surgical excision under local anesthesia for quick symptomatic relief and histopathologic diagnosis was discussed with the patient. The procedure was explained to the patient and informed, written consent was obtained. In the clinical setting and under local anesthesia, the mass was initially injected with lidocaine and epinephrine (1:100,00) and then completely excised via a CO_2_ surgical laser which resulted in no significant bleeding.

**Fig. 3:**
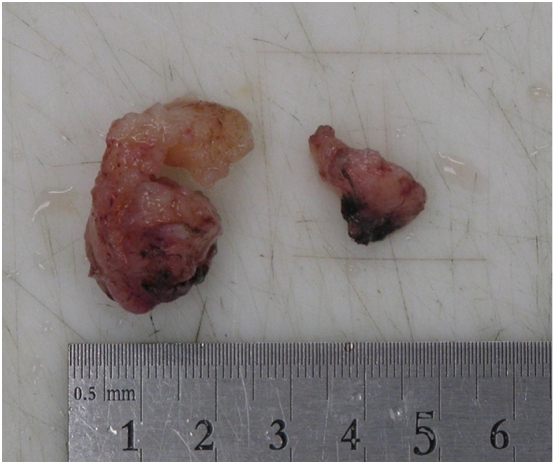
Gross appearance of the excised mass.

**Fig. 4:**
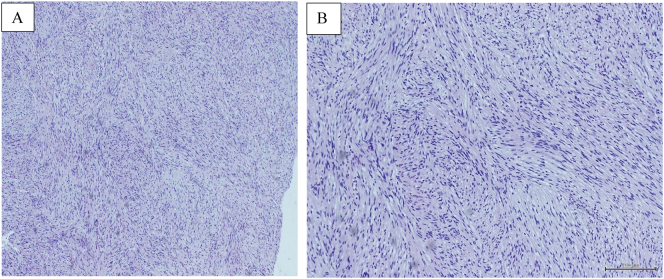
Histopathological findings. A. low magnification shows a hypovascular tumor with intersecting fascicles of spindle cell proliferation with no areas of necrosis identified (hematoxylin and eosin staining-4×). B. High magnification shows bland spindle cells with focal cellular palisades with no atypia or mitosis (hematoxylin and eosin staining-2×).

Gross histopathological analysis revealed a firm, pinkish tan mass measuring 30 mm in the maximum dimension with intact mucosal surface (Fig. 3). Microscopically, the mass was composed of biphasic cells with intersecting fascicles of moderately cellular spindle cells (Antoni A) plus a low representation of a hypocellular component (Antoni B). Verocay bodies were found (Fig. 4).

**Fig. 5:**
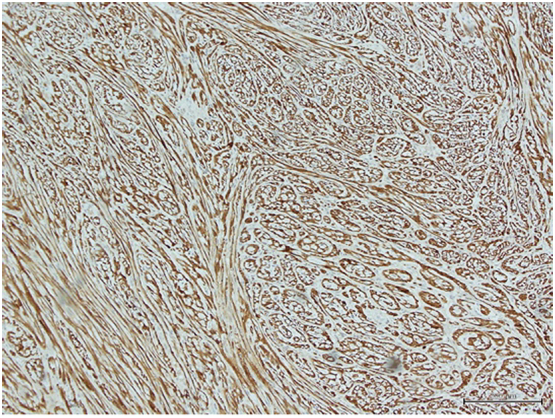
Immunohistochemical findings: neoplastic cells show diffuse immunoreactivity for S100 (20×).

Immunohistochemical (IHC) stains were performed. The neoplastic cells revealed strong immunoreactivity for S-100 protein, vimentin and CD31 (Fig. 5). Glial fibrillary acidic protein (GFAP), CD34, desmin, smooth muscle actin (SMA), cytokeratin AE1/AE3, and epithelial membrane antigen (EMA) were all negative. The tumor proliferative index (by Ki-67) was <1%.

**Fig. 6:**
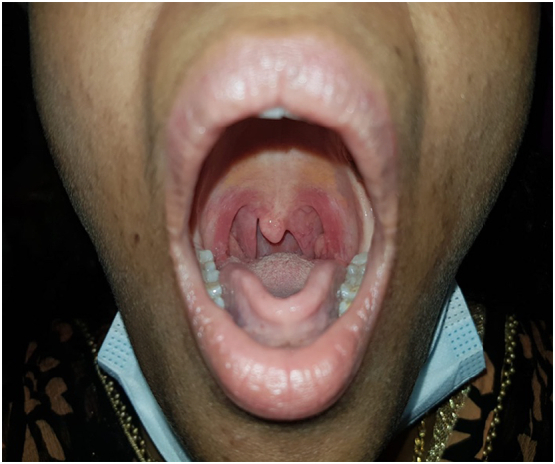
One-month post excision of the soft tissue lesion indicating good mucosal healing at the site of excision with no evidence of recurrence.

Based on the morphological and IHC profile findings, a final diagnosis of a benign soft palate schwannoma was made. The patient was completely asymptomatic during follow up appointment in our clinic after six months postoperatively, with healthy mucosa and no evidence of tumor recurrence during intraoral examination (Fig. 6).

## 3 Discussion

Schwannomas (neurilemmomas) are benign nerve sheath neoplasms that can arise from cranial, peripheral, or autonomic nerves that contain schwann cells. They were first described by Verocay in 1908 [8]. Schwannomas have predilection for head, neck, and surface flexors of the upper and lower extremities [2,3,4]. The tongue is the most common location of an intraoral schwannoma, and they rarely occur, as in the case presented, in the soft palate [5,6,7]. Regarding gender distribution, Williams et al. showed male predominance of this type of tumor, but Lucas et al. reported a greater incidence in females [8,9]. Schwannomas can occur at any age, although when present in the oral cavity they tend to be typically diagnosed between the ages of 25 and 55 [10]. The patient in this case was a 39-year-old female with a two-month history of a mass in her soft palate.

Intraoral and palatal schwannomas are frequently slow-growing, solitary lesions. Multiple nerve schwannomas require further assessment for Von-Reklinghousen disease (neurofibromatosis type 1), while bilateral vestibular schwannomas raise the suspicion of neurofibromatosis type 2 [11,12]. Despite the nervous system origin of these tumors, our patient presented with a solitary painless swelling on the lateral aspect of the palate with no genetic or familial tumor syndromes history. Similarly, most of the reported cases have asymptomatic, painless, slow-growing lesions. The clinical presentation of these tumors may differ based on the site, size, and involvement of nerves [13].

Clinically, these benign tumors are easily mistaken for other entities such as mesenchymal tumors (lipoma, hemangioma or leimyoma) or epithelial tumors (pleomorphic adenoma) [ 14], among other reasons since this is an infrequent tumor and is not usually suspected in the oral cavity. Moreover, based on the site of occurrence, benign traumatic neuroma, mucous retention cysts, fibroma, papilloma, and benign salivary gland tumors need to be ruled out [14]. Clinical differential diagnosis of the present case included salivary gland tumor and benign mesenchymal tumor based on the location, slow growth, and appearance of the lesion.

Imaging modalities such as CT are useful during the initial assessment to determine the extent of the tumor and narrow down the differential diagnosis. In our case, the CT scan revealed a well circumscribed mass without direct invasion, hemorrhage, or calcification, yet the clinical diagnosis was still suspicious and confounded with other differentials. Similarly, Kun et al. [15], were able to make a correct preoperative diagnosis in only 4 cases located in the neck of the 49 cases studied, they concluded that it is very difficult to make the diagnosis based on diagnostic imaging techniques as schwannoma is an infrequent tumor in the oral cavity, as was in our case.

Schwannomas have four major histologic phenotypes: conventional, plexiform, ancient, and cellular variants. The most common type is the conventional schwannoma that shows a biphasic architecture with two main regions: Antoni A and Antoni B. Antoni A regions are more cellular with palisading nuclei surrounded by eosinophilic areas (Verocay bodies), whereas Antoni B regions consist of less cellular areas with a predominant loose and/or myxomatous stroma [12,16]. In our case, the histopathological examination revealed an unencapsulated lesion with the characteristic Antoni A area with Verocay body and Antoni B areas. No atypia or mitosis was identified. This presented a typical histological examination of schwannoma, thus aiding in establishing the diagnosis.

IHC examination was essential for confirming the diagnosis of schwannoma, and protein S-100 was the first protein to be immunostained during the evaluation of a suspected peripheral nerve tumor. Diffuse staining with CD-34 is rarely seen in schwannomas, as it shows some focal staining in noncellular (Antoni B) regions. Schwannomas also stain positively for Leu-7antigen, GFAP, and vimentin. In our case, using IHC examination with S-100 protein and vimentin revealed intense positivity in the cells of the mass, confirming its neural origin [12].

Schwannomas are benign tumors rarely undergoing malignant transformation [15]. Complete surgical excision is the treatment of choice, and schwannomas generally do not recur if they are completely excised. However, it has been reported that a schwannoma has recurred after excision [16,17]. In our case, we completely resected the lesion using CO_2_ surgical laser under local anesthesia in the clinic. After six months of follow-up, there was no evidence of recurrence (Fig. 6).

## 4 Conclusion

A proper multi-disciplinary approach is required to provide the best standard of care in case of head and neck tumors. Therefore, combined morphological and immunohistochemical investigations are necessary to attend such tumors and reach a diagnosis of schwannoma, like in our case.

Complete surgical excision is the treatment of choice providing unlikely local recurrences.

## Ethical approval

The case report was approved by the institutional review board at King Hamad University Hospital in Al Muharraq, Bahrain.

## Funding sources

This research did not receive any specific grant from funding agencies in the public, commercial, or not-for-profit sectors.

## CRediT authorship contribution statement

Alhanouf Alhedaithy: Writing - Original Draft

Anwar Alsayed: Writing - Review & Editing

Khalid Al-sindi: Writing - Review & Editing

Waleed Janahi: Conceptualization, Resources, Supervision

## Guarantor

Alhanouf Alhedaithy

## Consent

Written informed consent was obtained from the patient for publication of this case report and accompanying images. A copy of the written consent is available for review by the Editor-in-Chief of this journal on request.

## Provenance and peer review

Not commissioned, externally peer-reviewed.

## Declaration of competing interest

None.
